# Making sense of health information technology implementation: A qualitative study protocol

**DOI:** 10.1186/1748-5908-5-95

**Published:** 2010-11-29

**Authors:** Rebecca R Kitzmiller, Ruth A Anderson, Reuben R McDaniel

**Affiliations:** 1 School of Nursing, Duke University, 307 Trent Drive, Durham, NC 27502, USA; 2 Department of Management Science and Information Systems, McCombs School of Business, The University of Texas at Austin, 1 University Sta B6000, Austin TX 78712-0201, USA

## Abstract

**Background:**

Implementing new practices, such as health information technology (HIT), is often difficult due to the disruption of the highly coordinated, interdependent processes (*e.g.*, information exchange, communication, relationships) of providing care in hospitals. Thus, HIT implementation may occur slowly as staff members observe and make sense of unexpected disruptions in care. As a critical organizational function, sensemaking, defined as the social process of searching for answers and meaning which drive action, leads to unified understanding, learning, and effective problem solving -- strategies that studies have linked to successful change. Project teamwork is a change strategy increasingly used by hospitals that facilitates sensemaking by providing a formal mechanism for team members to share ideas, construct the meaning of events, and take next actions.

**Methods:**

In this longitudinal case study, we aim to examine project teams' sensemaking and action as the team prepares to implement new information technology in a tiertiary care hospital. Based on management and healthcare literature on HIT implementation and project teamwork, we chose sensemaking as an alternative to traditional models for understanding organizational change and teamwork. Our methods choices are derived from this conceptual framework. Data on project team interactions will be prospectively collected through direct observation and organizational document review. Through qualitative methods, we will identify sensemaking patterns and explore variation in sensemaking across teams. Participant demographics will be used to explore variation in sensemaking patterns.

**Discussion:**

Outcomes of this research will be new knowledge about sensemaking patterns of project teams, such as: the antecedents and consequences of the ongoing, evolutionary, social process of implementing HIT; the internal and external factors that influence the project team, including team composition, team member interaction, and interaction between the project team and the larger organization; the ways in which internal and external factors influence project team processes; and the ways in which project team processes facilitate team task accomplishment. These findings will lead to new methods of implementing HIT in hospitals.

## Background

Hospital-based health information technology (HIT) implementation research is needed to identify reproducible strategies to eliminate barriers to HIT use and promote its adoption and integration [1]. We found few studies of HIT implementation, and this absence may contribute to the slow and inconsistent adoption of HIT observed in hospitals [2]. This study will address two weaknesses identified in the literature on hospital-based HIT implementation: the absence of evidence about strategies to improve implementation and how to construct and manage project teams tasked with HIT implementation.

In this study, we will prospectively examine a multidisciplinary project team as it prepares to implement a HIT system in a tertiary care hospital. Due to a lack of literature on project teamwork specific to HIT implementation, we rely on the general literature about hospital-based project teamwork. We will use sensemaking to explain the social processes embedded in large scale organization change [3-5], and qualitative methods to achieve the following aims: describe and compare sensemaking across multidisciplinary project teams whose members differ in terms of hierarchical role and discipline; describe how the sensemaking of multidisciplinary project teams changes over time; describe how multidisciplinary project teams' sensemaking influences the actions taken; and identify team member behaviors that facilitate or inhibit sensemaking of a multidisciplinary project team.

HIT implementation literature falls into three categories: anecdotal case reports, effectiveness research, and research describing HIT impact in clinical settings. First, the majority of hospital HIT implementation literature is anecdotal and lacks systematic evidence for sound implementation interventions [6,7]. Second, HIT efficacy studies often discuss lessons learned; however these lessons were explanations of findings, rather than empirical observations [8,9]. Finally, generalizability of HIT impact studies is hampered by methodological concerns [10,11]. The majority of studies used retrospective, self reported data, focused mainly on HIT system users, usually physicians, and evaluated a single type of HIT system, such as provider order entry. Thus, best implementation methods remain largely unknown.

HIT impact studies identified unanticipated social effects, such as reallocated work [12], interrupted work processes [11,13], altered information exchange, communication patterns, and interpersonal relationships [11-15], and in some cases, patient harm [10,11]. Studies have also found that hospital staff member's perspectives about HIT processes for, and outcomes of, implementation varied by organizational identity [16], role [17], and work unit [9], causing variation in action. Care providers who perceived HIT as a threat, resisted using the HIT system [18-20]. Those who saw HIT as a benefit to patient care, on the other hand, used the system and advocated for system improvements [20,21]. Thus, care providers varied implementation experiences combined with differing expectations, objectives, and needs may contribute to the slow and uneven adoption of HIT in hospitals.

Project teams have not been well studied, even though they are responsible for implementing HIT. Project teamwork is a popular strategy that hospitals use to create change [22]. To develop solutions and anticipate consequences of change, hospitals populate teams with members with different experience, skill, and knowledge [23]. Diverse members bring new information to the team and they provide connections with others in the organization [24,25]. Thus, effective teamwork facilitates collaboration, coordinated effort, and task accomplishment [13]. Studies show that teams are usually better problem solvers than individuals perhaps because they represent the combined input of all members, or because team member interactions facilitate learning associated with shared expertise and social interaction [26-29].

During HIT implementation projects, hospitals need access to various knowledge and skills to uncover interdependencies and critical expectations, and to determine actions. Research on healthcare project teams noted that diverse membership and positive interpersonal interaction was associated with team innovativeness and positive organizational outcomes [25,28,30-32]. However, studies found that diverse perspectives also created conflict that was linked to poor team performance [28]. It appears necessary to carefully manage relationships between people to achieve benefits of diversity. Interpersonal interaction and diversity of team membership, therefore, are an important focuses of the proposed study; specifically, we focus on sensemaking processes of the team.

### Theoretical framework: sensemaking

Sensemaking, a social process of searching for answers and meaning, drives the actions people take [33]. Sensemaking occurs through verbal discourse between hospital staff members. Whether planned or unplanned, change challenges people's ability to understand what is happening, to anticipate what will happen, and to know what steps to take [5,33,34], suggesting that sensemaking processes may be more important than decision-making processes for successful change. Because HIT implementation in hospital settings does not occur in a linear fashion and includes unpredicted, unexpected outcomes, implementation team members cannot expect to make optimal decisions [11,15,35]. They are forced to make 'good enough' choices and adjust as new information becomes available and understanding of circumstances changes [13]. When compared to traditional linear, process-focused perspectives on HIT-related change, such as decision making and diffusion of innovation [36,37], sensemaking may help us to better manage project team actions because it is a process that accounts for new information as events unfold and for social interaction and construction of meaning [38-41].

Research on sensemaking in hospital studies suggests several things. Organizational role, such as nurse, physician, or manager, influences the sense that staff members make of events [42-44]. The sense that hospital staff members make influences their choices and actions [45-47]. Through discourse with other staff members, hospital staff members construct the meaning of information and events and shape and reshape their understanding as events unfold and new information becomes available [46-48]. Project teamwork provides a formal mechanism for enhancing sensemaking. Through dialog, team members share varying perspectives on team tasks, construct the meaning of events as the HIT is implemented, and take action in response to evolving meaning. Through sensemaking, team members define what is happening, jointly revise their understanding, learn, and problem solve, setting the course for HIT implementation [42,44,49]. This view of sensemaking and the review of literature on project teams, thus serves as the basis for our methodological choices. Refer to Additional file 1 for additional reference material used in developing this study.

## Methods

### Design

We propose a qualitative, longitudinal multiple case study through which we will examine the evolving sensemaking of three multidisciplinary HIT project teams using direct observation and organizational document review. We will follow the activities of these teams throughout the pre-implementation phase of the project. We defined this period as the time between team formation and the first time the HIT is used by hospital staff in the provision of care [50]. Through our choice of methods, we plan to address the following four weaknesses in prior research on hospital HIT implementation, project teams and sensemaking: retrospective data collection; reliance on self-reported perceptions of HIT implementation; focus on single participant identity; and focus on single work units.

Following Institutional Review Board approval, we will contact the Chief Nursing Officer and Chief Information Officer to obtain permission to conduct the study. As an incentive, a consultation summarizing findings of the study with recommendations for future project teams will be provided to the organization and to the case study participants. Following a protocol described by Utley-Smith *et al. *[51], the consultation will serve as a method of disseminating research findings directly to study participants in the form of a summary of research findings and some recommendations related to teamwork strategies for more effective sensemaking. Knowledge participants gain during the consultation may validate study participants' project experience, influence decisions to participate in future projects, and enhance participants' IT implementation skills [52]. Subjects in prior research have reported that they perceived a direct benefit from such consultations and recommendations [51].

### Setting

This study will be conducted within a single, academic, tertiary care hospital in the southeastern United States. Consisting of 834 beds in 33 nursing units, the hospital has a highly complex, interdependent care environment where changes in care practices, such as HIT implementation, may result in unexpected consequences. The hospital decided to implement an HIT system, an electronic nursing documentation system, in its 33 nursing units. Because of the anticipated impact of this system, the hospital is forming a multidisciplinary project team comprised of nine sub-teams. Each sub-team will be tasked with a different aspect of the HIT project and staffed with a cross-section of clinical disciplines and functional business team members. Using selection criteria described below, each sub-team selected for inclusion will represent a single case. We anticipate that project team members will have little history of working together; thus, the unique knowledge each member brings to the team's tasks may be largely unknown by other members of the team and team management processes will be necessary.

### Sample selection

#### Selecting case study teams

Prior research suggests that team members' perspectives on HIT implementation may differ based upon their departmental affiliation, professional training, organizational role, and hierarchical level [4,41-43]. Further, a team's roles and responsibilities may shape the discourse, meaning, and actions taken during the project [41]. Thus, to capture how sensemaking is influenced by team member diversity, we will select sub-teams of the larger project implementation team for in-depth case study using two criteria: the sub-team has a broad scope of project responsibility within the larger project team and its members have different social identities. Three of nine project implementation sub-teams meet the criteria of broad responsibility and diverse membership and thus will be included in-depth case study: the executive team, the communication team, and the implementation team. The executive team (n = 9) will include administrative and clinical executives and directors from multiple departments, and has a broad scope in that it will provide resources for the project and ensure alignment of project goals with organizational strategic goals. The communication team (n = 11) will include administrative, clinical, and technical directors, managers, and staff representing many organizational levels, and has a broad scope in that it will produce all organizational communication about the project including minutes, articles, video, and web-based documents. The implementation team (n = 31) will include directors, managers, and front-line staff from nursing units, pharmacy, information technology, and hospital education, also representing many organizational levels. This team has a broad scope in that it will collect information about care practices, identify unit level information and care needs, and recommend modifications to the system in support of those needs.

The six sub-teams that will not be selected for in-depth case study include the steering committee, the neonatal development team, the psychiatric development team, the device selection team, the training team, and the informatics team. These teams will have narrower scopes of responsibility (*e.g.*, selecting equipment), or their membership will be homogenous (*e.g.*, all psychiatric nurses). To understand how the overall project is unfolding across the nine teams, however, we will collect published minutes from meetings held by the six teams not directly observed to include in analysis of documents. Further, during case study sub-team meetings, an update on the work of all nine teams will be summarized and presented. During the executive, communication, and implementation team meetings, we will capture this information in the field notes. Together, these documents and field notes will provide us with information about events and actions of other teams that we do not directly observe.

### Measurement

#### Sensemaking

Sensemaking will be measured qualitatively using direct observations of the executive, communication, and implementation teams; and project document review. This approach will capture multiple perspectives and rich data on HIT implementation events [13,39,41]. We derived a set of sensemaking behaviors from a literature review [5,33,42,53,54], which we evaluated in a preliminary study, and used to developed an observation guide (Additional file 2: Appendix A [55]) intended to capture sensemaking and subsequent actions. We anticipate that through discourse in team meetings, members will share their unique knowledge (*e.g.*, care processes within a department), their perspective on the HIT implementation, and their interpretation of information and events [56] that will then direct their actions [45]. The observation guide will also facilitate documenting the actions the teams plan to take and their anticipated results as well as the teams' reflections on the actions taken. Questions on the observation guide included the following: What information do participants share and how do they share it (*e.g.*, past experience, information from others, hypothetical scenarios)? What interpretations, labels, and conclusions do team members express? What new ideas, decisions or proposed actions will be taken and by whom? What form does the discussion take? And, how do team members interact with each other?

The document review guide (Additional file 2: Appendix B) is designed to capture written discourse where the project team formally records and/or shares information with external constituents about the team's goals and actions taken related to the HIT implementation. Data collection will include date obtained, description of the document, date of event or contact associated with the document, significance of the document, and a brief summary of the contents.

### Participant demographic data

Team member demographic data (Additional file 2: Appendix C) will be obtained by self-report at the time when participants are introduced to the study. Data collected will include current job title, current unit of assignment, tenure in their profession, tenure in the organization, tenure on the unit of assignment, highest educational level, highest educational level in the profession, technology experience, gender, age, and ethnicity/race. These data will be used to explore variations in sensemaking because studies indicate that these are individual characteristics that are likely to influence sensemaking [40,43,45,48].

### Data collection procedures

#### Direct observation

We will directly observe team meetings and activities (*e.g.*, training sessions) throughout the study. During observations, we will observe and manually record verbal communications between team members, using field notes and jottings [57]. These notes will be typed directly into a laptop versus being handwritten on paper and transcribed at a later time [57]. We will also document observations, such as seating arrangement, note passing, and eye rolling. We will audio record the meetings to support the field notes and listen to the tapes to verify that the field notes accurately capture communications; the recordings will not be transcribed verbatim. All data will be tagged with date and time to capture emerging trends. Meetings will generally occur once a month and last approximately 60 to 90 minutes. Direct observations occur during regularly scheduled meetings pose minimal burden to participants. Field notes, jottings, and audio recordings are tantamount to meeting minutes. Electronic field notes will be formatted and imported into AtlasTI.

### Documents

Documents related to the project (*e.g.*, articles) and project records (such as meeting minutes, presentations, policies and procedures, and flyers), will be maintained by the HIT project team in a Lotus notes database, published to the hospital intranet for review by hospital staff members, and published in organizational periodicals and newsletters. These documents are produced by the committee and reflect the way in which they wish to represent their work to external constituents. We will access documents electronically or in printed form from the intranet, and add them to the study database. Document date will be used to facilitate placement in and retrieval from the study database. Once formatted, documents will be imported into AtlasTI and summarized following the guide (Additional file 2: Appendix B). Documents will serve to corroborate and augment evidence from direct observation, or to contradict observational evidence [42,57].

### Participant demographic data

Participant consent for use of demographic data will be obtained after we provide a review of the nature of the study, participant's role, confidentiality, and the associated risk/benefits of participation. Participants will complete the survey tool described earlier (Additional file 2: Appendix C). As new team members are added, we will follow the demographic data collection procedure. The survey will take approximately 15 minutes to complete, posing minimal burden to participants. The demographic data will be entered into Microsoft Excel tables and accessed with SAS (v. 9.1) for analysis.

### Data analysis

We will use qualitative analysis procedures recommended by Crabtree and Miller [57]. Our research team contains experts in health informatics; organizational cooperation and fairness; and organizational sensemaking and learning. As we develop hypotheses for each research aim, we will conduct research team discussions to uncover bias and propose alternate perspectives on emerging themes.

### Code development

From the literature on sensemaking, we have developed an *a priori *set of codes (Additional file 2: Appendix D). Coding reduces the data so that the data remain manageable, facilitating data clustering and laying a foundation for further analysis [58]. Through iterative review and ongoing discussion between RK and RA, we will refine the definitions of each *a priori *code. When a segment of text does not fit an existing code, we will ask, 'What's going on here?' 'What triggered this participant action?' 'What follows this participant action?' 'How might sensemaking explain what is happening?' Through this open-coding technique, we will further develop our codes. We will develop decision rules and definitions to guide the categorization of data, and record these in the electronic codebook [57,58]. To minimize the loss of meaning that may occur when reducing data, we will record all data transition steps and retain original raw data, including meeting audio recordings, until the study is complete.

First, we will read the entire field note or document to get a sense of the whole and create an initial memo to capture our emerging impressions [58]. In a second reading, we will code units of text that described sensemaking events using our *a priori *codes. We will then create a second memo, summarizing initial ideas about the field note, documenting areas that need follow up [58]. Coded units will be sorted into categories and subcategories and analyzed for recurrent themes.

To address our research aims, we will use within-case and between-case analyses. To describe and compare sensemaking across multidisciplinary project teams, data will be analyzed for each case study sub-team so that we can gain a rich understanding of the sensemaking of each individual team [59]. In the cross-case analysis, for this aim, we will organize each team's sensemaking themes into three separate data matrices and compare across teams to establish similarities and differences among the teams. Because the three project teams' members differ in professional and organizational identity, it is likely that the teams will differ in terms of the sense they make of new information and project events. To describe how the sensemaking of temporary multidisciplinary project teams changes over time, we will analyze themes in temporal sequence. Since sensemaking is shaped by experience, it is likely that sensemaking of the project teams' members will shift following significant events. To describe how multidisciplinary project team's sensemaking influences the actions taken by the teams, we will organize the data matrices by the actions of each team in order to identify the antecedents and consequences of these actions. Finally, to identify which team member behaviors facilitate and or inhibit the sensemaking of a multidisciplinary project team, we will use open coding guided by the literature on project teams. Some examples of codes may include respect or openness to ideas. The coded data will be analyzed for themes that explain how team member behaviors either facilitate or inhibit team sensemaking.

### Assuring rigor

We will use several established strategies to assure confirmability, dependability, and credibility [57,58] in qualitative data collection and analysis. These are briefly described in Table 1. We will log all study material in a Microsoft Access database using a date/time/source stamp to facilitate access to these materials. This database will serve as the basis for an audit trail.

**Table 1:** Strategies for Ensuring Rigor

Criteria	Strategies to assure criteria are met
Confirmability: unrecognized researcher biases are controlled	RK and RA (and later the research team) serve as the check and balance for uncovering assumptions and suggesting rival hypotheses. Member checks will be used to confirm findings.

Dependability: candidate performance remains consistent over time	Guides will be used for all data collection. RK and RA will meet bi-weekly to review data collection and refine techniques. An electronic code book will be used to track all data transformations. An audit trail will be established. RK and RA will read and each code at least 50% of the field notes and compare coding. We will discuss and come to agreement about codes and interpretations.

Credibility: results are plausible and authentic	Triangulation of data from multiple sources: direct observation (multiple healthcare disciplines and organizational hierarchical levels) and documents. Member checks will be used to confirm findings.

## Discussion

This study appears to be the first to prospectively examine a multidisciplinary HIT implementation project team and its sub-teams. Hospitals often form project teams to provide a formal mechanism for sharing different perspectives on events, in this case, an HIT implementation, and developing solutions to implementation issues. The project team in this study represents a huge organizational investment in that more than 100 people will be involved in the project. Rather than using traditional, mechanistic models for studying HIT implementation in hospitals, we propose an innovative perspective--sensemaking--that reveals embedded social processes that shape large scale organization change. Effective sensemaking facilitates team members' understanding of what is happening, their learning, their problem solving, and, ultimately, the actions they take (or do not take) with regard to system implementation [40]. Prior research linked these activities to successful HIT implementation in non-hospital settings [4,40,60,61]. This study will: identify HIT implementation issues within the complex hospital environment and how team members deal with roadblocks and unexpected events; and describe the link between team member social interaction and implementation actions. These findings will lead to new methods of managing multidisciplinary project teams and implementing HIT in hospitals.

### Strengths and limitations

We recognize several limitations inherent in our design choices. Our study will be conducted in a single, large-scale academic hospital, thus generalizability of our findings to other types of healthcare organizations may be limited. We will neither interview individual project team members about their project team experience nor will we observe their interactions with people outside of the teams. However, because the sense the project team makes of ongoing implementation events is dependent upon verbal exchanges, we believe the choice to limit our observations to project team activities, such as meetings, will allow us to describe important discourse in sensemaking of HIT implementation. Finally, all project sub-teams will not be directly observed. However, we will note how our selected teams are keeping team members informed of other aspects of the overall project, and we will include documents from excluded sub-teams in our analysis.

Through our methodological choices, we aim to enrich the project team and hospital-based HIT implementation literature. Unlike many other studies, ours focuses on the people responsible for HIT implementation and will capture the interpretations and actions of a diverse group of project team members. Rather than relying on participant perceptions of events and potentially unreliable, retrospective data collection methods, the prospective case study design captures:

1. Key antecedents and consequences of ongoing, evolutionary, social process of implementing HIT.

2. Key internal and external factors that influence project teams including team composition, team member interaction, and interaction between project teams and the larger organization.

3. Key ways in which internal and external factors actually influence project team processes.

4. Key ways in which project team processes facilitate team task accomplishment.

The resulting in-depth, rich description of HIT implementation will facilitate determining how sensemaking differs among project teams, how sensemaking develops over time, what information and events teams respond to, what meaning is constructed, and what actions result from that meaning. Thus, this study will make a significant contribution to advancing our understanding of how project teams function within the complex hospital care environment and bring about organizational change.

## Competing interests

The authors declare that they have no competing interests.

## Authors' contributions

RK designed the study and drafted the manuscript. RA and RM guided study design and read and revised the manuscript. All authors read and approved the final manuscript.

## Supplementary Material

Additional file 1 **Additional reference material used in developing the background and significance for the study**. Click here for file

Additional file 2 Appendix A (direct observation guide); Appendix B (document guide); Appendix C (participant demographic survey tool); and Appendix D (*a priori *code list) Click here for file
