# Supplementary material for: Making sense of health information technology implementation: A qualitative study protocol
Source: Implement Sci. 2010 Nov 29;5:95. doi: 10.1186/1748-5908-5-95 (PMC3001692; doi:10.1186/1748-5908-5-95)
Supplement: Additional file 1 — Additional reference material used in developing the background and significance for the study. [file 1748-5908-5-95-S1.PDF]

Bibliography

Aarts J, Doorewaard H, Berg M: **Understanding implementation: The case of a computerized physician order entry system in a large dutch university medical center.** *J Am Med Inform Assoc* 2004, **11:**207-216.

Ancona DG, Caldwell DF: **Bridging the boundary: External activity and performanc in organizational teams.** *Adm Sci Q* 1992, **37:**634-665.

Ash JS, Sittig DF, Dykstra RH, Guappone K, Carpenter JD, Seshadri V: **Categorizing the unintended sociotechnical consequences of computerized provider order entry.** *Int J Med Inf* 2007, **76 Suppl 1:**21-27.

Ash JS, Sittig DF, Poon EG, Guappone K, Campbell E, Dykstra RH: **The extent and importance of unintended consequences related to computerized provider order entry.** *J Am Med Inform Assoc* 2007, **14:**415-423.

Baer M, Frese M: **Innovation is not enough: Climates for initiative and psychological safety, process innovations and firm performance.** *Journal of Organizational Behavior* 2003, **24:**45-68.

Bates DW, Teich JM, Lee J, Seger D, Kuperman GJ, Ma'Luf N, Boyle D, Leape L: **The Impact of Computerized Physician Order Entry on Medication Error Prevention.** *J Am Med Inform Assoc* 1999, **6:**313-321.

Berg M: **Patient care information systems and health care work: a sociotechnical approach.** *Int J Med Inf* 1999, **55:**87-101.

Berner ES, Detmer DE, Simborg D: **Will the Wave Finally Break? A Brief View of the Adoption of Electronic Medical Records in the United States.** *J Am Med Inform Assoc* 2005, **12:**3-7.

Bossen C: **Test the artefact - Develop the organization - The implementation of an electronic medication plan.** *Int J Med Inf* 2007, **76:**13-21.

Cannon MD, Edmondson AC: **Failing to learn and learning to fail (intelligently): how great organizations put failure to work to innovate and improve.** *Long Range Plann* 2005, **38:**299.

Choo CW, Johnston R: **Innovation in the knowing organization: a case study of an e-commerce initiative.** *Journal of Knowledge Management* 2004, **8:**77.

Cohen D, McDaniel R, Crabtree B: **A practice change model for quality improvement in primary care practice.** *J Healthc Manag* 2004, **49:**155 - 168.

Coopey J, Keegan O, Emler N: **Manager's innovations as 'sense-making'.** *British Journal of Management* 1997, **8:**301-315.

Crabtree BF, Miller WL, Tallia AF, Cohen DJ, DiCicco-Bloom B, McIlvain HE, Aita VA, Scott JG, Gregory PB, Stange KC, McDaniel RR, Jr.: **Delivery of clinical preventive services in family medicine offices.** *Annals of Family Medicine* 2005, **3:**430-435.

Crosson JC, Stroebel C, Scott JG, Stello B, Crabtree BF: **Implementing an electronic medical record in a family medicine practice: Communication, decision making, and conflict.** *Annals of Family Medicine* 2005, **3:**307-311.

Cummings JN: **Work Groups, Structural Diversity, and Knowledge Sharing in a Global Organization.** *Management Science* 2004, **50:**352.

De Dreu CKW, Weingart LR: **Task versus relationship conflict and team effectiveness: A meta-analysis. .** *J Appl Psychol* 2003, **88:**741-7449.

DesRoches CM, Campbell EG, Vogeli C, Zheng J, Rao SR, Shields AE, Donelan K, Rosenbaum S, Bristol SJ, Jha AK: **Electronic Health Records' Limited Successes Suggest More Targeted Uses.** *Health Aff* 2010, **29:**639-646.

Devine DJ, Philips JL: **Do smarter teams do better: A meta-analysis of congitive ability and team performance.** *Small Group Research* 2001, **32:**507-532.

Edmondson AC: **Framing for learning: Lessons in successful technology implementation.** *Calif Manage Rev* 2003, **45:**34-+.

Edmondson AC: **Learning from mistakes is easier said than done: Group and organizational influences on the detection and correction of human error.** *The Journal of Applied Behavioral Science* 1996, **32:**5.

Edmondson AC: **Speaking up in the operating room: How team leaders promote learning in interdisciplinary action teams.** *Journal of Management Studies* 2003, **40:**1419 - 1452.

Fleeman BF: **A case study of diversity in making sense of a change intervention: Lessons learned with insights from complexity science.** *Ph.D.* The University of Texas at Austin, 2002.

Ford EW, McAlearney AS, Phillips MT, Menachemi N, Rudolph B: **Predicting computerized physician order entry system adoption in US hospitals: Can the federal mandate be met?** *Int J Med Inf* 2008, **77:**539-545.

Franklin BD, O'Grady K, Donyai P, Jacklin A, Barber N: **The impact of a closed-loop electronic prescribing and administration system on prescribing errors, administration errors and staff time: a before-and-after study.** *Quality and Safety in Health Care* 2007, **16:**279-284.

Ganster DC, Williams S, Poppler P: **Does training in problem solving improve the quality of group decisions?** *J Appl Psychol* 1991, **76:**479-483.

Georgiou A, Ampt A, CreswiCk N, Westbrook JI, Braithwaite J: **Computerized Provider Order Entry-What are health professionals concerned about? A qualitative study in an Australian hospital.** *Int J Med Inf* 2009, **78:**60-70.

Gersick CJG: **Revolutionary change theories: A multilevel exploration of the punctuated equilibrium paradigm.** *Acad Manage Rev* 1991, **16:**10-36.

Goldstein MK, Coleman RW, Tu SW, Shankar RD, O'Connor MJ, Musen MA, Martins SB, Lavori PW, Shlipak MG, Oddone E, et al: **Translating Research into Practice: Organizational Issues in Implementing Automated Decision Support for Hypertension in Three Medical Centers.** *J Am Med Inform Assoc* 2004, **11:**368-376.

Haas MR: **Knowledge gathering, team capabilities, and project performance in challenging work environments.** *Management Science* 2006, **52:**1170-1184.

Hackman R (Ed.). **Groups That Work and Those That Don't**. San Francisco: Jossey-Bass; 1990.

Harrison MI, Koppel R, Bar-Lev S: **Unintended Consequences of Information Technologies in Health Care--An Interactive Sociotechnical Analysis.** *J Am Med Inform Assoc* 2007, **14:**542-549.

Hendry J: **Strategic decision making, discourse, and strategy as social practice.** *Journal of Management* 2000, **37:**955-977.

Hirst G, Mann L: **A model of R&D leadership and team communication: The relationship with project performance.** *R&D Management* 2004, **34:**147-160..

Horbar JD, Rogowski J, Plsek PE, Delmore P, Edwards WH, Hocker J, Kantak AD, Lewallen P, Lewis W, Lewit E, et al: **Collaborative quality improvement for neonatal intensive care. NIC/Q Project Investigators of the Vermont Oxford Network.** *Pediatrics* 2001, **107:**14-22.

Howell JM, Shea CM: **Individual differences, environmental scanning, innovation framing, and champion behavior: key predictors of project performance.** *Journal of Product Innovation Management* 2001, **18:**15-27.

Ilgen DR, Hollenbeck JR, Johnson M, Jundt D: **Teams in organizations: From input-process-output models to IMOI models.** *Annu Rev Psychol* 2005**:**517-543.

Jensen TB, Aanestad M: **Hospitality and hostility in hospitals: a case study of an EPR adoption among surgeons.** *European Journal of Information Systems* 2007, **16:**672-680.

Jensen TB, Aanestad M: **How healthcare professionals "make sense" of an electronic patient record adoption.** *Information Systems Management* 2007, **24:**29-42.

Jones MR: **"Computers can land people on Mars, why can't they get them to work in a hospital?" - Implementation of an Electronic Patient Record System in a UK Hospital.** *Methods Inf Med* 2003, **42:**410-415.

Karsh BT, Escoto KH, Beasley JW, Holden RJ: **Toward a theoretical approach to medical error reporting system research and design.** *Appl Ergon* 2006, **37:**283-295.

Kuperman GJ, Bobb A, Payne TH, Avery AJ, Gandhi TK, Burns G, Classen DC, Bates DW: **Medication-related clinical decision support in computerized provider order entry systems: A review.** *J Am Med Inform Assoc* 2007, **14:**29-40.

Lapointe L, Rivard S: **Getting physicians to accept new information technology: insights from case studies.** *Can Med Assoc J* 2006, **174:**1573-1578.

Leonard D, Sensiper S: **The role of tacit knowledge in group innovation.** *Calif Manage Rev* 1998, **40:**112-132.

Lemieux-Charles L, McGuire WL: **What do we know about health care team effectiveness? A review of the literature.** *Med Care Res Rev* 2006, **63:**263-300.

Lemieux-Charles L, Murray M, Baker GR, Barnsley J, Tasa K, Ibrahim SA: **The effects of quality improvement practices on team effectiveness: A mediational model.** *Journal of Organizational Behavior* 2002, **23:**533.

Liang T-P, Liu C-C, Lin T-M, Lin B: **Effect of team diversity on software project performance.** *Industrial Management and Data Systems* 2007, **107:**636-653.

Lyerla F: **Design and implementation of a nursing clinical decision support system to promote guideline adherence.** *Cin-Computers Informatics Nursing* 2008, **26:**227-233.

Maitlis S: **The social process of organizational sensemaking.** *Acad Manage J* 2005, **48:**21.

McDaniel RR, Jr.: **Management strategies for complex adaptive systems: Sensemaking, learning, and improvisation.** *Performance Improvement Quarterly* 2007, **20:**21-42.

Menachemi N, Brooks RG: **Reviewing the benefits and costs of electronic health records and associated patient safety technologies.** *J Med Syst* 2006, **30:**159-168.

Menachemi N, Randeree E, Burke DE, Ford EW: **Planning for hospital IT implementation: A new look at the business case.** *Biomedical Informatics Insights* 2008, **1:**29-44.
Miller SJ, Wilson DC: **Perspectives on organizational decision-making.** In *The Sage Handbook of Organization Studies.* 2nd edition. Edited by Clegg SR, Hardy C, Lawrence TB, Nord WR. London: Sage; 2006: 469-484
Nightingale PG, Adu D, Richards NT, Peters M: **Implementation of rules based computerised bedside prescribing and administration: intervention study.** *BMJ* 2000, **320:**750-753.
Paré G, Elam JJ: **Introducing Information Technology in the Clinical Setting: Lessons Learned in a Trauma Center.** *Int J Technol Assess Health Care* 1998, **14:**331-343.
Patterson ES, Cook RI, Render ML: **Improving patient safety by identifying side effects from introducing bar coding in medication administration.** *J Am Med Inform Assoc* 2002, **9:**540-553.
Poissant L, Pereira J, Tamblyn R, Kawasumi Y: **The impact of electronic health records on time efficiency of physicians and nurses: a systematic review.** *J Am Med Inform Assoc* 2005, **12:**505-516.
Proenca EJ: **Team dynamics and team empowerment in health care organizations.** *Health Care Manage Rev* 2007, **32:**370-378.
Reagans R, Argote L, Brooks D: **Individual Experience and Experience Working Together: Predicting Learning Rates from Knowing Who Knows What and Knowing How to Work Together.** *Management Science* 2005, **51:**869.
Sims DE, Salas E, Burke CS: **Promoting effective team performance through training.** In *The Handbook of Group Research and Practice.* Edited by Wheelan SA. Thousand Oaks, CA: Sage; 2005: 407-425
Sittig DF, Krall M, Kaalaas-Sittig J, Ash JS: **Emotional Aspects of Computer-based Provider Order Entry: A Qualitative Study** *J Am Med Inform Assoc* 2005, **12:**561-567.
Stevenson KB, Barbera J, Moore JW, Samore MH, Houck P: **Understanding Keys to Successful Implementation of Electronic Decision Support in Rural Hospitals: Analysis of a Pilot Study for Antimicrobial Prescribing.** *Am J Med Qual* 2005, **20:**313-318.
Schofield RF, Amodeo M: **Interdisciplinary teams in health care and human services settings: Are they effective?** *Health Soc Work* 1999, **24:**210-219.
Soffe SM: **The properties of sensemaking: A case study of meaning development during organizational change.** *Ed.D.* The George Washington University, 2002
Solberg LI, Hroscikoski MC, Sperl-Hillen JM, Harper PG, Crabtree BF: **Transforming medical care: case study of an exemplary, small medical group.** *Annals of Family Medicine* 2006, **4:**109-116.
Stewart GL: **A meta-analytic review of relationships between team design features and team performance.** *Journal of Management* 2006, **32:**29-55.
Stoop AP, Berg M: **Integrating quantitative and qualitative methods in patient care information system evaluation: guidance for the organizational decision maker.** *Methods Inf Med* 2003, **42:**458-462..

Thomas JB, Clark SM, Gioia DA: **Strategic sensemaking and organizational performance: linkages among scanning, interpretation, action, and outcomes.** *Academy of Management Journal* 1993, **36:**239-270.

Tucker AL, Edmondson AC: **Why hospitals don't learn from failures: Organizational and psychological dynamics that inhibit system change.** *Calif Manage Rev* 2003, **45:**55.

Tucker AL, Nembhard IM, Edmondson AC: **Implementing new practices: An empirical study of organizational learning in hospital intensive care units.** *Management Science* 2007, **53:**894.

Vaughn TE, McCoy KD, BootsMiller BJ, Woolson RF, Sorofman B, Tripp-Reimer T, Perlin J, Doebbeling BN: **Organizational predictors of adherence to ambulatory care screening guidelines.** *Med Care* 2002, **40:**1172-1185.

Wilkens R, London M: **Relationships between climate, process, and performance in continuous quality improvement groups.** *J Vocat Behav* 2006, **69:**510.
