# Supplementary material for: Making sense of health information technology implementation: A qualitative study protocol
Source: Implement Sci. 2010 Nov 29;5:95. doi: 10.1186/1748-5908-5-95 (PMC3001692; doi:10.1186/1748-5908-5-95)
Supplement: Additional file 2 — Appendix A (direct observation guide); Appendix B (document guide); Appendix C (participant demographic survey tool); and Appendix D (a priori code list) [file 1748-5908-5-95-S2.DOC]

Appendix A

Direct Observation Guide: Team Meetings

| Activity | Meeting System Testing Training |
| --- | --- |
| Subteam | Executive Communication Implementation |
| Length of activity |  |
| Who was present? |  |
| Form of the discussion (e.g., one-way, two-way, multidirectional, briefing, training) | |
| What information do participants share? (e.g., past experience, information from others, hypothetical scenarios) | |
| Describe categorization, “new ideas,” interpretations, conclusions, decisions, or proposed actions to be taken and by whom | |
| Describe how participants interact with each other (e.g., heated debate, developing storytelling, silence, avoiding eye contact, respectful) | |

Appendix B

*A-priori* Sensemaking Codes

| **Team Discourse** | |
| --- | --- |
| Categorizing | Providing a classification, grouping with others based on similar properties. For example, **“**this seems like a hardware issue.” [33, 34] |
| Cue | Information (signals or encounters) that is discrepant or inconsistent with what is expected [38];  information that is singled out and embellished [5] |
| Declaring | Making controversial statements to invite discussion. For example, “You mean we bought this system and now we don’t think it will improve patient care?” |
| Events | Providing an example case. May come from a team member’s experience, the team’s experience, the larger organizations’ experience; or be an imagined situation or case [61] |
| Framing | A bounded map of meanings; providing a particular view of organized action[46]; |
| Interpreting | Providing meaning about an event or about what is said [33] |
| Iterative dialog | Back and forth dialog between multiple team members about ideas, developing impressions, active reflecting, adding cues to build a story or throwing out ideas for problem solving [48] |
| Labeling | Providing meaning of an experience or situation through the use of a distinct cognitive representation. For example, “This project has gone south”, implying a bad outcome or turn for the worse. [38] |
| Pausing the flow | Making statements that stop the dialog and potentially cause members to reflect on the conversation and ongoing events and information [33, 34] |
| Probing | Asking for more information or further explanation. These become cues when they hint or direct attention to needed functionality or possible problems. |
| Questioning | Challenging the validity of information or conclusion as it was presented. For example, “I have not had the same experience.” |
| Reflecting | Examining the outcomes of action and or experimentation; what this means for the individual or for the team [34] |
| Reframing | Offering an alternative explanation in terms of known events or circumstances. For example, “this isn’t just about the nurses, what about the patients’ needs?” [38] |
| Validating | Checking in, confirming understanding (restating/summarizing) or that knowledge is represented correctly |
| **Qualities of Interpersonal Interaction** | |
| Respectful | Demonstrating regard and valuing input of other team members [28] |
| Disrespectful | Demeaning or denigrating contributions of other team members [28] |
| Group Process | Actions designed to engage team members: inviting feedback; seeking clarification; confirming understanding; promoting voice; promoting information sharing; summarizing plans, actions, next steps [28] |
| **Team Activities** | |
| Action | Any tasks the team is currently doing [38] |
| Action-Experimenting | New tasks the team specifically undertakes to learn and innovate [38] |
| Action-suggesting | Tasks one or more members of the group proposes to undertake [38] |
| Boundary spanning | Sharing information, experiences, and details from sources external to the team [30] |

Appendix C

Document Summary Form*

| Document # |  | |
| --- | --- | --- |
| Date obtained: |  | |
| Name or description of document: | | |
| Event or Contact, if any, with which document is associated: | | |
|  | | |
| Date of event or contact: | |  |
|  | | |
| Significance or importance of the document: | | |
| Brief summary of contents: | | |
| *Adapted from Miles and Huberman (1994) | | |

Appendix D

Staff Demographic Form

The following questions are about you and your work on the nursing and patient care documentation system implementation team. Please complete the questions as accurately as possible.

This research study uses a coding system to protect your identity as a study participant and ensure confidentiality of the information you provide.

Please return this document to Rebecca Kitzmiller or send it by campus mail in the envelope provided: DUMC 3322.

| Your name: |  |
| --- | --- |

1. What profession best describes you?

| (1) | Dietician |  | (5) | Respiratory Therapist |  |  | | |
| --- | --- | --- | --- | --- | --- | --- | --- | --- |
| (2) | Pharmacist |  | (6) | Social worker |  |  | | |
| (3) | Physician |  | (7) | Other, please indicate: |  | | | |
| (4) | RN, Nurse |  |  |  | | |  |  |

| 2. How long have you worked in the profession listed above? |  |
| --- | --- |

3. What best describes your highest level of education in the profession listed above? Please check one.

| (1) | High school |  | (4) | Bachelor's degree |  |  |
| --- | --- | --- | --- | --- | --- | --- |
| (2) | Diploma |  | (5) | Master's degree |  |  |
| (3) | Associate degree |  | (6) | Other, please indicate: |  | |

4. What best describes your over all level of education? Please check one.

| (1) | High school |  | (4) | Bachelor's degree |  |  |
| --- | --- | --- | --- | --- | --- | --- |
| (2) | Diploma |  | (5) | Master's degree |  |  |
| (3) | Associate degree |  | 6) | Other, please indicate: |  | |

5. Which unit / service line /department do you most often work?

| (1) | General Surgery |  | (19) | Orthopedics |  | (36) | Accreditation | |  |  |
| --- | --- | --- | --- | --- | --- | --- | --- | --- | --- | --- |
| (2) | 2100 |  | (20) | 6100 |  | (37) | Customer Support Services | |  |  |
| (3) | 2200 |  | (21) | 6300 |  | (38) | Education Services | |  |  |
| (4) | 2300 |  | (22) | Heart |  | (39) | Finance | |  |  |
| (5) | 3100 |  | (23) | 7100 |  | (40) | Nursing Informatics | |  |  |
| (6) | 3200 |  | (24) | 7200 |  | (41) | Nutrition Services | |  |  |
| (7) | 3300 |  | (25) | 7300 |  | (42) | Pastoral Care | |  |  |
| (8) | Neurosciences |  | (26) | General Medicine |  | (43) | Performance Services | |  |  |
| (9) | 4100 |  | (27) | 8200 |  | (44) | Pharmacy | |  |  |
| (10) | 4200 |  | (28) | 8300 |  | (45) | Procurement | |  |  |
| (11) | 4300 |  | (29) | Step-down |  | (46) | Psychiatry | |  |  |
| (12) | Pediatrics |  | (30) | 7800 |  | (47) | Public Relations | |  |  |
| (13) | 5100 |  | (31) | 8100 |  | (48) | Risk Management | |  |  |
| (14) | 5200 |  | (32) | Oncology |  | (49) | Social Work Services | |  |  |
| (15) | 5300 |  | (33) | 9100 |  | (50) | Technology Education | |  |  |
| (16) | Pediatrics/Neonate |  | (34) | 9200 |  | (51) | Women’s Services | |  |  |
| (17) | 5400 |  | (35) | 9300 |  | (52) | Other; please indicate: |  | | |
| (18) | 5500 |  |  |  |  |  |  | |  |  |

| 6. How long have you worked in this unit / service line / department? |  |
| --- | --- |

7. What best describes your current job title?

| (1) | Staff |  | (8) | Associate Chief Nursing Officer | |  |  |
| --- | --- | --- | --- | --- | --- | --- | --- |
| (2) | Nurse Educator |  | (9) | Associate Chief Information Officer | |  |  |
| (3) | Nurse Manager |  | (10) | Chief Finance Officer | |  |  |
| (4) | Manager |  | (11) | Chief Operating Officer | |  |  |
| (5) | Clinical Operations Director |  | (12) | Chief Nursing Officer | |  |  |
| (6) | Director |  | (13) | Chief Information Officer | |  |  |
| (7) | Associate Operating Officer |  | (14) | Other, please indicate: |  | | |

| 8. How long have you worked in the job title listed above? |  |
| --- | --- |
| 9. How many years have you worked for Duke University Hospital? |  |

10. What best describes your level of technology experience?

| (1) | Excellent: I teach others | |  |  |
| --- | --- | --- | --- | --- |
| (2) | Pretty good: I can figure it out | |  |  |
| (3) | Adequate: I know the basics | |  |  |
| (4) | None: I don’t use computers | |  |  |
| (5) | Other; please indicate |  | | |

11. Are you:

| (1) | Female? |  |
| --- | --- | --- |
| (2) | Male? |  |

12. What is your age?

| (1) | Less than 20 |  | (5) | 35-44 |  |
| --- | --- | --- | --- | --- | --- |
| (2) | 20-24 |  | (6) | 45-54 |  |
| (3) | 25-34 |  | (7) | 55 and over |  |

13. What best describes your race and/or ethnicity? Please check as many as apply.

| (1) | American Indian or Alaskan Native |  |
| --- | --- | --- |
| (2) | African-American |  |
| (3) | Asian or Pacific Islander |  |
| (4) | Hispanic |  |
| (5) | White, not of Hispanic origin |  |
| (6) | I do not wish to provide this information |  |
